# Exergaming in older adults: A scoping review and implementation potential for patients with heart failure

**DOI:** 10.1177/1474515113512203

**Published:** 2013-11-06

**Authors:** Leonie Verheijden Klompstra, Tiny Jaarsma, Anna Strömberg

**Affiliations:** 1 Department of Social and Welfare studies, Faculty of Health Science, Linköping University, Sweden; 2 Department of Medical and Health Science, Division of Nursing Science, Faculty of Health Science, Linköping University, Sweden

**Keywords:** Exergame, active video game, elderly, exercise, virtual reality

## Abstract

**Background:**

Physical activity can improve exercise capacity, quality of life and reduce mortality and hospitalization in patients with heart failure (HF). Adherence to exercise recommendations in patients with HF is low. The use of exercise games (exergames) might be a way to encourage patients with HF to exercise especially those who may be reluctant to more traditional forms of exercise. No studies have been conducted on patients with HF and exergames.

**Aim:**

This scoping review focuses on the feasibility and influence of exergames on physical activity in older adults, aiming to target certain characteristics that are important for patients with HF to become more physically active.

**Methods:**

A literature search was undertaken in August 2012 in the databases PsychInfo, PUBMED, Scopus, Web of Science and CINAHL. Included studies evaluated the influence of exergaming on physical activity in older adults. Articles were excluded if they focused on rehabilitation of specific limbs, improving specific tasks or describing no intervention. Fifty articles were found, 11 were included in the analysis.

**Results:**

Exergaming was described as safe and feasible, and resulted in more energy expenditure compared to rest. Participants experienced improved balance and reported improved cognitive function after exergaming. Participants enjoyed playing the exergames, their depressive symptoms decreased, and they reported improved quality of life and empowerment. Exergames made them feel more connected with their family members, especially their grandchildren.

**Conclusion:**

Although this research field is small and under development, exergaming might be promising in order to enhance physical activity in patients with HF. However, further testing is needed.

## Introduction

Regular daily exercise is recognized as important from both the perspective of primary and secondary prevention in cardiac disease.^1^ Since heart failure (HF) is a frequent discharge diagnosis it is important to look for any opportunity to improve outcomes. In a recent position paper by the Heart Failure Association of the European Society of Cardiology, the importance of increased activity and exercise in cardiac patients’ cardiovascular conditions was advocated.^2^ More specifically, guidelines on the treatment of HF also recommend regular physical activity and structured exercise training, since they improve exercise capacity, quality of life, do not adversely affect left ventricular remodelling and may reduce mortality and hospitalization in patients with mild to moderate chronic HF.^2^

Physical impairment is described as a significant problem in older adults with HF and exercise capacity in patients with HF is approximately 50–75% of normal age and gender predicted values.^3^ Several studies have shown that both home-based exercise (often distance walking)^4,5^ and hospital based^6–8^ is safe and beneficial for patients with HF. The findings from a meta-analysis (ExTraMatch collaborative) suggested that patients randomized to physical fitness were less likely to be admitted to hospital and had a better prognosis.^9^ Although the HF-ACTION (Heart Failure: A Controlled Trial Investigating Outcomes of Exercise Training) trial did not find significant reductions in the primary end point of all-cause mortality or hospitalization, this study showed a modest improvement in exercise capacity and mental health in patients who exercised. The main limitation in this study was the poor adherence to the prescribed training regimen (only 30% after 3 years).^10^ Adherence to exercise recommendations in patients with HF is low and low adherence has a negative effect on the clinical outcomes, such as HF readmission and mortality.^11^ There are many factors that influence adherence in general, and more specifically adherence to exercise. Therefore, it is important to search for alternative approaches to motivate patients with HF to exercise.^2,12,13^

A scoping review of health game research showed a constant growth over recent years and positive progress towards adapting new technology in specialized health contexts. Most health game studies included physical activity (28%) using so-called exergames (games to improve physical exercise).^14^ A meta-analysis of energy expenditure (EE) in exergaming showed that playing exergames significantly increased heart rate, oxygen uptake and EE compared to resting, and may facilitate light- to moderate-intensity physical activity promotion.^15^ The use of these exergames might also be an opportunity for patients with HF to increase their physical activity at home and encourage them to exercise more regularly, especially those who may be reluctant to engage in more traditional forms of exercise, such as going to the gym or taking a walk outside.

A recent review of exergaming for adults with systematic disabling conditions showed that most participants in research with exergames are male and stroke survivors.^16^

There are no studies on exergaming in patients with HF, and therefore this scoping review was conducted. The purpose of a scoping review is to identify gaps in the existing literature, thereby highlighting where more research may be needed. In contrast to a systematic review, it is less likely to seek to address very specific research questions nor, consequently, to assess the quality of the included studies.^17^ This scoping review focuses on the feasibility and influence of exergames on physical activity in older adults, aiming to target certain characteristics that are important for patients with HF in order to become more physically active. These characteristics were safety, balance, cognition and experiences.

The research questions to be answered were:

- Is exergaming feasible and safe for older adults?
- Do exergames influence physical activity in older adults?
- Do exergames influence balance in older adults?
- Do exergames influence cognition in older adults?
- What are the experiences of older adults playing exergames?

## Methods

**Figure 1.**
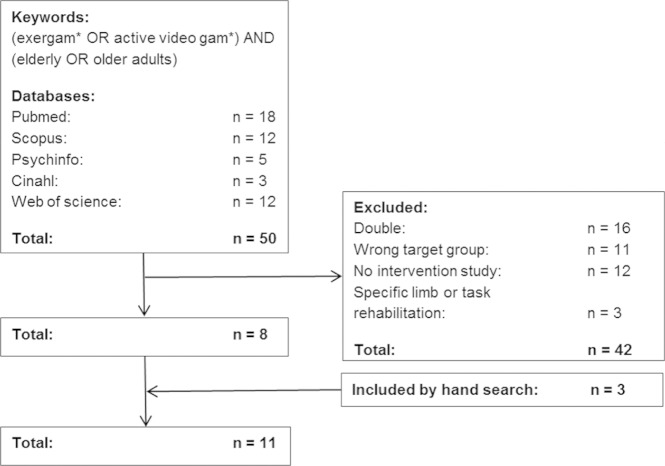
Inclusion of studies in the review.

A literature search was undertaken in August 2012 in the international online bibliographic databases PsychINFO, PubMed, Scopus, Web of Science and CINAHL. The keywords used were: exergame OR active video game AND elderly OR older adults (Figure 1). In addition to searching the databases, the references of relevant publications were checked. Articles that met the following criteria were included in the review: focusing on the influence of exergames or active video games on older adults’ physical activity (mean age research population ≥ 50 years old) and written in English. Articles were excluded if they were focused on specific limbs or at improving specific tasks or if they did not describe any intervention (e.g. articles on the development of an exergame, descriptive studies). The title and/or abstracts of the studies were scanned for the study objective, study population, exergame platform, training procedure, measurements and main conclusions (Tables 1 and 2).

**Table 1.** Characteristics of the studies.

Author continent	Study objective	Design methodological quality^18^	Research population	Exergame platform	Training procedure	Key outcome measurements	Key results
1. Agmon et al. (2011)^19^ America	To determine the safety and feasibility of exergaming to improve balance in older adults	Pre-post VIII	Seven community-dwelling older adults with impaired balance, mean age (SD) 84 (5), four women	Nintendo Wii	3 months (three times a week for 30 minutes) with at least five home visits with individualized instructions	*Balance*: BBS *Mobility impairment and gait speed*: Timed 4-Meter Walk Test *Exercise enjoyment*: PACES *Feasibility and safety*: Semi-structural weekly phone calls and written logs, and semi structural interview at post-test	Improved BBS, Timed 4-Meter Walk Great enjoyment after exergaming Expressed improved balance in daily activity and desire to play with their grandchildren Two games had to be modified to ensure safety, no participants experienced a fall during the intervention
2. Anderson-Hanley et al. (2012)^20^ America	To compare the cognitive benefits of cybercycling with traditional stationary cycling	RCT III	79 community-dwelling older adults EXP: *n*=38, mean age (SD), 76 (10), 33 women CON: *n*=41, mean age (SD), 82 (6), 29 women	Cybercycle	*1^st^ month* EXP and CON three times a week (45 minutes) familiarization with biofeedback stationary biking *2^nd^ Month* EXP: Cybercycle, three times a week (45 minutes) CON: Traditional biking + placebo training, three times a week (45 minutes)	*Cognitive assessment*: Color Trials 2-1 difference score, Stroop C, Digit Span Backwards *Physiologic*: iDXA (GE Lunar, Inc.). HUMAC Cybex Dynamometer (CSMI Solutions, Inc.), insulin, glucose *Assessment of exercise behavior*: ACLS-PAQ, accelerometer, ride behaviors recorded with bike computer *Neuroplastic assessment*: Fasting morning plasma, BDNF levels	Improved cognitive performance in executive function and neuroplasticity. EXP 23% relative risk reduction in clinical progression to mild cognitive impairment Effort and fitness no factors behind differential cognitive benefits in EXP
3. Anderson-Hanley et al. (2011)^21^ America	To examine the effect of virtual social facilitation and competitiveness on exercise effort in exergaming older adults	Subgroup analyses IV	14 community-dwelling older adults (eight low competiveness, six high competiveness), age range 60–99, 13 women	Cybercycle	1 month (2–3 rides a week), cybercycling with virtual competitors	*Competitiveness*: Competitiveness Index *Exercise effort*: 10 second interval by cybercycle sensors	High competiveness older adults had a higher riding intensity than low competiveness older adults
4. Chuang et al. (2006)^22^ Asia	To evaluate the effect of a virtual “country walk” on the number of sessions necessary to reach cardiac rehabilitation goals in patients undergoing coronary artery bypass grafting	RCT III	20 male outpatients who had bypass surgery EXP: *n*= 10, mean age (SD) 66 (15) CON: *n*=10, mean age (SD) 64 (10)	Cyberwalking	EXP: 3 months (twice a week for 30 minutes) cyberwalking CON: 3 months (twice a week for 30 minutes) training on treadmill	*Cardiorespiratory testing*: The Naughton protocol *Maximum work rate*: Treadmill speeds and grades	Number of sessions required to reach target heart rate and target VO2 was lower in EXP than CON Maximum workload EXP was higher than CON
5. Maillot et al. (2011)^23^ Europe	To assess the potential of exergame training in cognitive benefits for older adults	RCT III	32 community-dwelling older adults, mean age (SD) 73 (3), 27 women EXP: *n*=16, mean age (SD) 73 (4) CON: *n*=16, mean age (SD) 73 (3)	Nintendo Wii	EXP: 14 weeks (24 times 1 hour) exergaming CON: No training, no contact	*Physical impact of the training*: The functional fitness test *Executive control tasks, visuospatial tasks, processing-speed task*: The cognitive battery	EXP had a higher game performance, physical function, cognitive measured of executive control and processing speed than CON No differences between EXP and CON on visual spatial measures
6. Rand et al. (2008)^24^ Asia	To investigate the potential of using exergaming for the rehabilitation of older adults with disabilities	Pre-post VIII	Study 1: 34 young adults, mean age (SD) 26 (5), 17 women Study 2: 10 older adults without a disability, mean age (SD) 70 (6), six women Study 3: 12 individuals age range 50–91, seven women	IREX VR system Sony PlayStation EyeToy	Study 1: Played the two exergame platforms for 180 seconds in addition to 60 seconds of practice, in total 40 minute 1 time session in a clinic Study 2: Played three exergames on the Sony PlayStation 180 seconds in addition to 60 seconds of practice at home Study 3: Played two exergames on the Sony PlayStation 180 seconds in addition to 60 seconds of practice at home, clinic or hospital	*Sense of presence*: PQ *Feedback of exergames*: SFQ *Physical effort*: Borg’s Scale of Perceived Exertion *Performance*: Monitored by scores in each exergame *System usability*: SUS	No difference in sense of presence IREX and EyeToy in young adults High enjoyment exergaming in the research population EyeToy seems less suitable for acute stroke patients
7. Rosenberg et al. (2010)^25^ America	To assess the feasibility, acceptability, and short-term efficacy and safety of a novel intervention using exergames for SSD	Pre-Post VIII	19 community-dwelling adults with SSD, mean age (SD) 79 (9), 13 women	Nintendo Wii	12 weeks (three times a week for 35 minutes) exergaming with guidance *Follow up*: 12 weeks after intervention	*Mood*: QIDS, BAI *Health-Related QoL*: MOS SF-36 *Cognitive functioning*: RBANS *Rating individual Wii Sports on enjoyment*: Likert scale from 1 (least) to 7 (most) *Wii adherence: L*og of activity for 12 weeks	Decrease in depressive symptoms Increase in mental related QoL and cognitive function Adherence 84% No major adverse events
8. Saposnik et al. (2010)^26^ America	Comparing the feasibility, safety, and efficacy of exergaming in rehabilitation versus recreational therapy (playing cards, bingo, or jenga)	RCT III	22 stroke patients, mean age (range) 61 (41–83), 14 women EXP: *n*=11, mean age (range) 67 (46–83) CON: *n*=11, mean age (range) 55 (41–72)	Nintendo Wii	EXP: 2 weeks (eight sessions of 60 minutes) exergaming CON: 2 weeks (eight sessions of 60 minutes) recreational therapy *Follow-up*: 4 weeks after intervention	*Feasibility*: Time tolerance and adaption to exergaming (total time receiving intervention) *Safety*: Proportion of patients experiencing intervention-related adverse events or any serious adverse events during the study period *Motor function*: WMFT	No serious adverse events No difference EXP and CON in symptoms No difference in feasibility between EXP and CON EXP had higher motor function than CON
9. Smith et al. (2012)^27^ Oceania	To develop and establish characteristics of exergaming in older adults	Pre-post VIII	Recruited from a pool of 44 community-dwelling older adults, mean age 79	DDR	One time session in a clinic	*Step responses*: USD DDR mat *Characteristics of stepping performance*: Purpose built software	Older adults are able to interact with DDR Stepping performance is determined by characteristics of game play such as arrow drift speed and step rate
10. Taylor et al. (2012)^28^ Oceania	To quantify EE in older adults playing exergames while standing and seated and to determine whether balance status influences the energy cost associated with exergaming	Pre-post VIII	19 community-dwelling adults, mean age (SD) 71 (6), 15 women	Nintendo Wii Xbox 360 Kinect	Played nine exergames, each for 5 minutes, in random order. Bowling and boxing were played both seated and standing	*EE*: Indirect calorimeter *Balance*: Mini-BESTest, ABC scale, TUG	EE exergaming result in light physical activity No difference EE Nintendo Wii and EE Kinect No difference EE exergaming sitting and standing No difference between EE or activity counts and balance status
11. Wollersheim et al. (2010)^29^ Oceania	To investigate the physical and psychological effect of exergaming	Pre-Post, Focus Groups VIII	11 older women who participated in community planned activity groups, mean age (SD) 74 (9)	Nintendo Wii	6 weeks (twice a week between 9–130 min each session) exergaming	*Body movements*: Accelerometer *Psychosocial effects*: Focus groups	EE increased with gameplay No difference in overall EE Results focus groups: Greater sense of physical, social and psychological well-being

ABC Scale: Activities Specific Balance Confidence Scale; ACLS-PAQ: Aerobics Center Longitudinal Study Physical Activity Questionnaire; BAI: Beck Anxiety Inventory; BBS: Berg Balance Scale; BDNF: Brain-derived Neurotrophic Growth Factor; CON: Control group; DDR: Dance Dance Revolution EE: Energy Expenditure; EXP: Experimental group; IREX: Interactive Rehabilitation and Exercise System; Mini-BESTest: Balance Evaluation Systems Test; PACES: Physical Activity Enjoyment Scale; PQ: Presence Questionnaire; QIDS: Quick Inventory of Depressive Symptoms; QoL: Quality of life; RBANS: Repeatable Battery for the Assessment of Neuropsychological Status; SD: Standard deviation; SFQ: Short Feedback Questionnaire; SSD: Subsyndromal depression; TUG: Timed up and go; USD: Universal Serial Bus; VO2: oxygen uptake; VR: virtual environment.

**Table 2.** Articles’ main conclusion.

Exergame platform	Description of exergame platform	Outcomes				
		Feasibility and safety	Physical activity	Balance	Cognition	Participants’ experiences
**Nintendo Wii**	Game computer with a wireless controller which detects movements in three dimensions through Bluetooth	Participants felt comfortable playing after five individualized training sessions^19^ Certain games were too difficult to play^19,29^ Adherence: 84–97.50%^23,25^ Practice resulted in improved performance on exergaming^23^ No serious adverse events^26^ Exergaming was feasible for stroke patients^26^	↑ EE^29^ ↑ Gait speed^19^ ↑ Physical status, especially cardiorespiratory fitness^23^ Exergaming resulted in light to moderate intensity range of activity^23,28^ ↑ Motor function^26^ No difference in EE exergaming while standing or sitting^28^	↑ Balance^19^ No relationship between EE or activity and balance status^28^	↑ Cognitive benefit^23,25^ ↑ Executive function^23^ ↑ Processing speed^23^	High level of enjoyment^19,23^ and would like to continue exergaming^23^ An experience that could be shared with the family, especially with grandchildren^19,29^ ↑ Mental related Quality of Life^25^ No increase in symptoms^26^ and decreased depression symptoms^25^ ↑ Sense of physical, social and psychological well-being^29^
**Dance Dance Revolution (DDR)**	Game computer with a dance mat including four step-sensitive target panels	Older adults were able to interact with the DDR^27^ Stepping performance was determined by characteristics of game play such as arrow drift speed and step rate^27^				
**Xbox 360 Kinect**	Game computer with a webcam-style add-on peripheral that enables players to interact without the need to touch a game controller		Exergaming resulted in light physical activity^28^			
**Sony PlayStation Eyetoy**	Game computer with a USB camera that translates body movements into a controller input	Less suitable for acute stroke patients^24^				High enjoyment and sense of presence exergaming^24^
**Cybercycling**	Enhanced stationary cycling using virtual tours		↑ EE than stationary cycling^20^		↑ Cognitive benefit, executive function compared to stationary biking^20^	Introduction of an on-screen competitor led to an increase in riding intensity for more competitive older adults, compared to less competitive, older adults^21^
**Cyberwalking**	Enhanced treadmill walking using virtual tours		↑ Max workload in cyberwalking than treadmill^22^ ↓ Number of sessions required to reach target heart rate and VO2 when cyberwalking compared to treadmill training^22^			Participants described cyberwalking as feeling immersed in the VR scene^22^

EE: energy expenditure; VR: virtual reality; VO2: oxygen uptake.

The methodological quality was evaluated by a classification system, which has previously been used in reviews on new health technology and medical procedures in health care (Table 3).^18^ In this scoping review the methodological quality of the studies did not determine inclusion or exclusion.

**Table 3.** Classification of study designs (18).

Level	Strength of evidence	Type of study design
**I**	Good	Meta-analysis of randomized controlled trials
**II**		Large-sample randomized controlled trials
**III**	Good to fair	Small-sample randomized controlled trials
**IV**		Non-randomized controlled prospective trials
**V**		Non-randomized controlled retrospective trials
**VI**	Fair	Cohort studies
**VII**		Case-control studies
**VIII**	Poor	Non-controlled clinical series, descriptive studies
**IX**		Anecdotes or case reports

## Results

A total of 50 articles were found in the databases. Sixteen articles were duplicated in the databases and 26 articles were excluded because they did not meet the inclusion criteria. Three additional articles were found through a manual search. Finally, a total of 11 articles were included (Figure 1).

### Methodological aspects of the studies

One study was published in 2006,^22^ one in 2008,^24^ three in 2010,^25,26,29^, three in 2011,^19,21,23^, and three studies were published in 2012.^20,27,30^ Four studies used a randomized design.^20,22,23,26^ Because of the low number of participants in each randomized study (20–63 participants), the evidence of these studies is good to fair (Table 3). Seven studies used a pre-posttest design without a control group.^19,21,24,25,27–29^ One study reported a subgroup analysis of a randomized control trial^21^ and one pre-posttest study reported results from focus group interviews.^29^ Only two studies used a longer follow-up period; 4 weeks^26^ and 12 weeks.^25^

### Research populations

The largest study in this review included 63 older adults,^20^ and the smallest study included seven older adults.^19^ The majority examined community-dwelling older adults.^19,25,28,29^ Three studies included patient populations. One study included 32 patients with cardiac disease,^22^ two studies included stroke patients, one included 12 stroke patients and 10 older adults without a disability^24^ and one study included 22 stroke patients.^26^ Nine studies included both men and women. In these studies the majority of the participants were female (between 57 and 93%).^19–21,23–27,30^ One study included only men (*n*=20)^22^ and one only women (*n*=11).^29^ The age range in the studies was 50–99 years old.

### Safety and feasibility of exergaming

The exergame platforms in the studies seem to be safe and feasible with none of the studies reporting adverse events. After having received instructions and familiarized themselves with the exergames, stroke patients had no problems playing them.^26^ In a study where a balance board was used (on which the player stands during training), two games had to be modified due to muscle pain or balance problems in order to be safe and feasible. In this study patients had no problems playing the games after five individualized training sessions.^19^ In one study including older women, there were difficulties playing some of the exergames on the Nintendo Wii, and this study reported that mastery of the exergame seemed to be an important factor when choosing a favorite game to play.^29^ The Sony PlayStation EyeToy was feasible for older adults and stroke patients. It was less suitable for acute stroke patients due to weak upper extremity, which made it difficult to interact with the exergame platform.^24^ Older adults were able to interact with the Dance Dance Revolution. A significant relationship was found between stepping performance and stimuli characteristics, but the stepping performance decreased as stimulus speed and step rate were increased.^27^ The adherence in exergaming was between 84 and 98%.^23,25,26^

### Physical activity in exergaming

Eight studies using different instruments measured outcomes in physical activity (Table 1). Playing the exergames resulted in more EE compared to rest and to sedentary computer gaming.^28^ No significant difference in EE was found in playing bowling and boxing on the Nintendo Wii while standing up compared to playing these games while seated.^28^ In addition, no difference in EE was found between the exergame platforms Nintendo Wii and Xbox Kinect.^28^ Playing the exergames resulted in an EE of light intensity exercise to moderate intensity activity.^23,28^ No significant correlation was found between EE or activity counts and balance status while bowling or boxing on the Nintendo Wii.^23^

Adding a virtual competitor in cybercycling increased the exercise effort among the more competitive exercisers.^21^ Cardiac patients who rehabilitated with cyberwalking had an increased workload and needed fewer sessions to reach their maximum heart rate and oxygen uptake, compared to a control group who had rehabilitation with only a treadmill.^22^

### Balance in exergaming

Three studies included balance as an outcome, using different instruments to measure this concept (Table 1). Participants experienced improved balance in daily activities after exergaming with the Wii Balance Board.^19^ One study showed that balance was not related to the amount of physical activity.^28^

### Cognition and exergaming

Cognitive change has been examined in three studies, measured by different instruments (Table 1). Participants had improved cognitive function in all of the three studies after exergaming,^20,23,25^ especially in executive function and processing speed.^23^ Cybercycling achieved better cognitive function than traditional exercises, using the same effort.^20^

### Experience in exergaming

Five studies included the experiences of participants who had used the exergame platform. The participants enjoyed playing the exergames^19,23,24^ and liked to continue using them.^23^ The studies do not report on preference based on age and gender. Participants who played exergames decreased in depressive symptoms (sustained at 12 week follow-up), and increased in Mental related Quality of Life^25^ and empowerment,^29^ measured with validated questionnaires (Table 1). They perceived health benefits in terms of greater ease of movements and psychosocial well-being.^29^ Within their family, the exergames allowed them to share experiences, which made them feel more connected with their family members, especially their grandchildren.^19,29^

## Discussion

Although this research field is still small and developing, we found that using exergame platforms might be a potentially effective alternative to facilitate rehabilitation therapy after illness and are suitable for use in older adults.

The studies showed that exergaming was safe and feasible, and could increase physical activity in elderly patients suffering from stroke and cardiac disease. The physical activity level increased while playing exergames, from light intensity exercise to moderate intensity activity. In four studies, exergaming resulted in positive outcomes in relation to balance and cognitive performance.^20,23,25,30^ In four studies, participants reported enjoyment in being active and one study resulted in a decrease of depressive symptoms.^19,23,24,29^ An important aspect of introducing exergaming to older adults is that a proper familiarization period is included and guidance is provided.

It will still be a challenge to find the most suitable exergame for a certain patient group. Although all games were found to be effective, some games were more strenuous than others and this might be important to consider when implementing or testing a certain exergame in a specific population. The commercial exergame platforms have the advantages that they are relatively cheap and health care providers have reported that the use of a commercial exergame platform (Nintendo Wii) provided purposeful and meaningful opportunities to promote well-being for older and disabled clients within a care and disability service for the elderly.^31^

This review is a first step to investigate the possibility of using an exergame platform to help patients with HF to adopt a more physically active lifestyle. The results of this review suggest that exergames increase physical activity in elderly individuals, stroke patients and cardiac patients, and could therefore be feasible and safe for patients with HF. However, further testing is needed. This review has some limitations, mainly the small sample sizes in the studies included in the review and the fact that most studies did not include a control group.

The findings of this review may have implications for both the current policy on delivery intervention programs that aim to increase physical activity, as well as the direction of future research. Further research, with a higher level of methodological quality and that examines the relative efficacy and costs of intervention programs aimed to enhance daily activity in non-health care settings, such as home settings, is needed. Also, a longer follow up period is needed to examine the long-term effects of these promising exergame platforms. Therefore, a RCT-study is planned to assess the influence of exergaming on exercise capacity in patients with heart failure (clinicaltrial.gov identifier: NCT01785121).
